# Automatic control of negative emotions: Evidence that structured practice increases the efficiency of emotion regulation

**DOI:** 10.1080/02699931.2014.901213

**Published:** 2014-03-31

**Authors:** Spyros Christou-Champi, Tom F. D. Farrow, Thomas L. Webb

**Affiliations:** ^a^ Department of Neuroscience, Unit of Academic Clinical Psychiatry, University of Sheffield, Sheffield, UK; ^b^ Department of Psychology, University of Sheffield, Sheffield, UK

**Keywords:** Implicit emotion regulation, Heart rate variability, Automatic regulation, Practice, Training intervention

## Abstract

Emotion regulation (ER) is vital to everyday functioning. However, the effortful nature of many forms of ER may lead to regulation being inefficient and potentially ineffective. The present research examined whether structured practice could increase the efficiency of ER. During three training sessions, comprising a total of 150 training trials, participants were presented with negatively valenced images and asked either to “attend” (control condition) or “reappraise” (ER condition). A further group of participants did not participate in training but only completed follow-up measures. Practice increased the efficiency of ER as indexed by decreased time required to regulate emotions and increased heart rate variability (HRV). Furthermore, participants in the ER condition spontaneously regulated their negative emotions two weeks later and reported being more habitual in their use of ER. These findings indicate that structured practice can facilitate the automatic control of negative emotions and that these effects persist beyond training.

Emotion regulation (ER) is the process by which people stay positive in the face of adversity, keep calm under pressure and prevent themselves from becoming overwhelmed by feelings such as disgust, anger or sadness. ER can lead to changes in the valence, intensity, time course and approach–avoidance tendencies associated with the activated emotion (Koole, 2009; Webb, Miles, & Sheeran, 2012) and can influence attention (Amir, Beard, Burns, & Bomyea, 2009), appraisal (Gross, 1998) and physiological responses, like heart rate (Denson, Grisham, & Moulds, 2011). ER is typically portrayed as a strenuous process (Koole, 2009) that aims to modulate spontaneous emotional responses (Duckworth, Bargh, Garcia, & Chaiken, 2002). Evidence suggests that effortful processes like ER are characterised by diffuse neural activity (Jansma, Ramsey, Slagter, & Kahn, 2001; Little, Klein, Shobat, McClure, & Thulborn, 2004) and supported by the cardiovascular system (i.e., increases in heart rate above those predicted by somatic requirements; Kennedy & Scholey, 2000; Turner & Carroll, 1985; Weis, 1986). It is not surprising, therefore, that effortful regulation makes extensive use of cognitive and physiological resources (Muraven, Tice, & Baumeister, 1998). For example, effortful ER compared to emotional acceptance was found to lead to poorer self-control in a subsequent stop-signal task requiring response inhibition (Alberts, Schneider, & Martijn, 2012).

ER can, however, also be implemented more automatically (Gyurak, Gross, & Etkin, 2011; Mauss, Bunge, & Gross, 2007). In contrast to effortful processes, automatic processes can be characterised by a range of features including being efficient, fast, spontaneous, stimulus driven, unconscious and relatively difficult to control. However, contemporary views suggest that these features should be investigated separately (Moors & De Houwer, 2006). In the present research, we focus primarily on the efficiency of ER. Therefore, “automatic” ER is taken to mean that people downregulate an unwanted emotional state using fewer resources and therefore decreasing the need for elevated cardiac outputs. This efficiency may occur because automatic processing is associated with a better connected neural network (where less neurons are active), as opposed to the more diffuse activity that characterises effortful processes (Jansma et al., 2001; Little et al., 2004). In short, automatic processes decrease the need for elevated cardiac outputs to supply active neural structures with glucose (Kennedy & Scholey, 2000; Turner & Carroll, 1985; Weis, 1986). This is consistent with evidence suggesting that automatic ER results in a lower heart rate when compared with effortful ER (Williams, Bargh, Nocera, & Gray, 2009).

## Capitalising on the benefits of automatic ER

More automatic forms of ER are likely to enable people to exert continuous control over their emotional responses because they are less resource intensive. The potential benefits of being able to regulate emotional responses in a relatively automatic fashion raise the question of whether individuals can develop strategies to capitalise on these benefits. The present research aimed to develop relatively automatic ER through the use of structured practice, a potential mechanism through which effortful processes such as ER can become more efficient (Bargh & Ferguson, 2000; Gyurak et al., 2011). Indeed, research on cognitive bias modification has reported that structured practice increases the efficiency with which participants directed their attention away from negatively valenced material (MacLeod, Koster, & Fox, 2009). However, mastering a single ER process or skill, like attentional control, does not necessarily ensure proficiency in other ER processes such as monitoring the need to regulate nor does it ensure proficiency in the application of ER strategies (Walden & Smith, 1997). Therefore, the present research examined the effects of training on the application of an ER strategy designed to help individuals to modify their emotional responses.

Previous research examining the effects of structured practice in reappraising negative events has produced encouraging results. For example, Schartau, Dalgleish, and Dunn (2009) trained some participants to reappraise negatively valenced film clips while another group watched the same film clips but were instructed to avoid regulating their emotions. Following training, both groups were asked to reappraise their emotional responses to further film clips. Participants who had practiced reappraisal showed lower levels of negative affect compared with those who were not trained. An additional study demonstrated that practice in reappraisal reduces the intrusion of negative memories over the subsequent week. However, Schartau et al. did not examine the impact of their intervention on physiological indicators of the efficiency of ER (e.g., cardiac responses), nor did they examine whether practice promoted efficient, habitual ER over time.

## The present research

We investigated whether structured practice can increase the immediacy and the efficiency of ER as measured by the time taken to regulate emotions and cardiac responses. Cardiac responses were measured in terms of variability in interbeat intervals (IBIs), indexed by heart rate variability (HRV). HRV refers to the degree to which the time interval between successive heart beats fluctuates. Neurophysiological models consider HRV to be a biomarker of successful ER (Thayer & Lane, 2009) and empirical studies have shown that effortful cognitive processing decreases HRV (Aasman, Mulder, & Mulder, 1987) while automatic cognitive processing increases HRV (Denson et al., 2011). This is in accord with literature suggesting that a decreased number of neurons are recruited after people have learned to complete a task in a more automatic fashion (Jansma et al., 2001; Little et al., 2004). Decreased neuronal activity reduces the need for cardiac acceleration (indexed by reduced HRV) in order to supply active neural populations with metabolic substrates (Kennedy & Scholey, 2000; Turner & Carroll, 1985; Weis, 1986). The aforementioned findings suggest that increases in IBI times (and thus HRV) reflect efficient task completion (Saling & Phillips, 2007) and therefore more automatic ER.

The present research asked participants to complete three training sessions over one week designed to increase the immediacy and efficiency with which they reappraised their emotional responses to images depicting body injuries (e.g., a mutilated toe). Previous research has shown that pictures depicting body injuries are associated with greater startle reflexes, as measured via blink magnitude, indicating their potency to act as aversive stimuli (Fairchild, Van Goozen, Stollery, & Goodyer, 2008). A follow-up session was conducted two weeks after the final training session. We hypothesised that training in the application of ER would result in faster ER (lower response latencies) and increased HRV during late but not early training trials, thus demonstrating both a practice effect and an increased efficiency over time. Two weeks later, we predicted that participants who had practiced using reappraisal would be more likely to spontaneously regulate their negative emotions and report being more habitual in so doing.

## METHOD

### Participants and design

Thirty participants were recruited from staff and students at a large university in the UK via email and flyers. Three participants were excluded from analyses due to not completing all sessions leaving 27 participants (18 females; *M*_age_ = 21.93, SD = 6.36 years; range = 18–46 years). Participants were randomly assigned to one of three conditions. In two of these conditions, participants received training but were given different instructions to follow: control instructions (*n* = 9) and ER instructions (*n* = 8). Participants in the third condition (*n* = 10) received no training and simply completed follow-up measures. Participants reported no history of psychiatric, neurologic and medical illness and the study was approved by the local ethics board.

### Procedure

#### Baseline emotional valence rating

**Figure 1.**
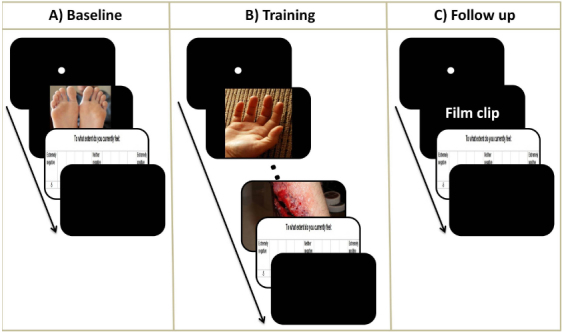
Schematic depiction of the paradigms, including baseline, training and follow-up phases. (A) Baseline paradigm. Participants were presented with 260 images (150 depicting body injuries and 110 depicting non-injured body parts). Each picture was preceded by the presentation of a fixation dot for 500 ms. Pictures were presented for 256 ms, followed by the self-report of emotional state that remained on-screen for three seconds. This was followed by a 500 ms inter-trial interval to take the total time for each baseline trial to four seconds. (B) Training paradigm. Between 0 and 9 pictures of normal, uninjured body parts were presented for one second preceding the presentation of the target (i.e., body injury) picture. Target pictures were presented for 10 seconds, followed by a presentation of the self-report of emotional state scale for five seconds. This was followed by a variable inter-trial interval to take the total time for each training trial to 25 seconds. (C) Follow-up paradigm. Participants were presented with five film clips lasting between 60 and 105 seconds. Each film clip was preceded by a presentation of a fixation dot for 500 ms and followed by the same self-report measure as that used during baseline and training which lasted for three seconds. This was followed by a variable inter-trial interval to take the total time for each follow-up trial to 109 seconds.

Before commencing training, participants provided a baseline measure of the impact of the pictures on their emotional state. Participants were presented with 150 images depicting body injuries as well as 110 images depicting non-injured body parts (Figure 1A). Seventy-five images depicting body injuries were taken from the International Affective Picture System (IAPS; *M*_Val_ = 2.13, SD = 0.60; possible range of responses = 1–9, 1–3 = negative valence; Lang, Bradley, & Cuthbert, 2005). The remaining pictures were created by editing pictures depicting non-injured body parts using Adobe Photoshop CS5 (Adobe Systems Inc., San Jose, CA). Participants viewed the images via the Presentation software (Neurobehavioural Systems Inc., Albany, CA), in a pseudorandomised order, seated approximately 50 cm from the screen. In line with research examining implicit emotion evaluation (e.g., Duckworth et al., 2002), each picture was presented for 256 ms to capture initial emotional responses while avoiding extensive conscious processing.

#### Self-reports of current emotional state

Following Schartau et al. (2009), participants indicated to what extent they felt either positive or negative on an 11-point scale anchored by “extremely negative” (1) and “extremely positive” (11). Participants’ emotional state was significantly more negative after they were exposed to the images (*M* = 3.33, SD = 0.90) than before (*M* = 8.38, SD = 1.50), *t*(15)=12.58, *p* < .001. The same was not true after exposure to images depicting non-injured body parts (*M* = 7.72, SD = 0.87), *t*(15)=1.46, *p* = .17.

#### Training

Following baseline evaluation, participants completed three training sessions, typically on alternating days. Participants were told via written instructions that during each trial they would be presented with a series of pictures, some of which depicted scenes designed to evoke negative emotions. Participants in the ER condition were instructed to reinterpret the content of the target pictures in order to alter their emotional responses (Gross, 1998) using the phrase “When seeing these injuries you must remind yourself that they are fake; they were created by the film industry using special effect technology to be used in movies”. Participants in the ER condition were told to press the “Enter” key when their emotional response to each negative image subsided. Participants in the control training condition were simply told: “When you see any injury, please press the ‘Enter’ key”.

Each training session comprised of 80 trials presented in a pseudorandom order. Each trial contained a maximum of 10 images (Figure 1B). Thirty trials contained 10 images depicting non-injured body parts presented for one second each. The remaining 50 trials presented participants with between 0 and 9 images depicting non-injured body parts followed by a picture depicting an injury. Pictures depicting body injuries could appear at any position ranging from the first to the tenth picture in order to avoid expectancy effects. Trials in which the picture depicting an injury was presented first always followed trials that contained no picture depicting an injury. Pictures depicting body injuries remained on-screen for 10 seconds to allow sufficient time for ER and were followed by the same self-report measure as that used during baseline, which served as a measure of ER success. No stimulus was presented after the self-report item and thus the inter-trial interval was varied such that each trial lasted for 25 seconds in total.

#### Cardiovascular effort exerted

HRV was collected using photoplethysmography (PPG) signal sensors attached to the left ear lobe. PPG belongs to a category of methods used to measure cardiac outputs that also includes electrocardiography (ECG) and pulse oximetry (Lu, Yang, Taylor, & Stein, 2009). To establish baseline HRV, participants were presented with a slideshow containing 30 images of neutral valence (*M*_Val_ = 5.2, SD = 0.3, possible range of responses 1–9, 4–6 = neutral valance) from the IAPS (Lang et al., 2005). Images remained on-screen for five seconds and the 30 images were repeated in a random order for five minutes. HRV was measured as the root mean square of successive differences (RMSSD) in the IBIs (Camm et al., 1996). This time-domain measure is correlated with measures reflecting high frequency components of the respiratory range (Goedhart, Van Der Sluis, Houtveen, Willemsen, & De Geus, 2007). It therefore indicates parasympathetic influences to the heart and thus decreases in cardiac responding. The output of the PPG sensor was digitally sampled and an online Butterworth low-pass filter, with a cut-off at 7 hz, was applied to remove any high frequency noise present in the signal. Artefact pre-processing was conducted on the IBI data following Goedhart et al.’ s (2007) guidelines. Change scores were computed subtracting baseline from training HRV. Therefore, positive scores represent increases in HRV (indexing decreased cardiovascular responding).

#### Follow-up

During the follow-up session, two weeks after the final training session, participants watched five film clips lasting between 60 and 105 seconds presented in a pseudorandom order (Figure 1C). The use of film clips during the follow-up allowed us to (1) examine whether training in ER influenced more sustained emotional responses, such as those induced by film clips (Westermann, Spies, Stahl, & Hesse, 1996) that are more ecologically valid than pictures (Gross & Levenson, 1995), and (2) reduce shared method variance and the likelihood that any effects of our training paradigm are task-specific (Holmes, Lang, & Shah, 2009). The film clips were obtained from Internet-based video-hosting sites and showed scenes including war violence, the infection of human tissue by fly larvae and the removal of a cyst from a person’s back. Following each film, participants rated their current emotional state on the same 11-point Likert scale used previously. No instructions were given regarding the application of ER as we were interested in the extent to which ER could be elicited relatively automatically by negative stimuli (Posner & Snyder, 1975).

In order to confirm that the film clips were successful in inducing negative emotions, we compared participants’ self-reported emotional state in the no-training condition before and after they were exposed to the film clips. As hypothesised, the valence of participants’ emotional state was significantly more negative after they were exposed to the film clips (*M* = 3.48, SD = 1.41) than before (*M* = 8.30, SD = 1.25), *t*(9) = 13.84, *p* < .001.

#### Habitual use of reappraisal

After watching the film clips, participants completed the self-report habit index (SRHI; Verplanken & Orbell, 2003) to measure the extent to which ER had become a habitual process. The SRHI comprises 12 items, including the history of behavioural repetition, the difficulty of controlling behaviour, lack of awareness and efficiency. Participants were provided with the stem—“Changing the way that I think about negative emotional situations in order to feel better is something”— and then responded to a number of statements (e.g., I do without thinking) on 11-point scales anchored by “strongly disagree” (1) to “strongly agree” (11). The 12 items showed high internal consistency (Cronbach’s alpha = 0.91).

#### Automatic use of reappraisal

Given that the 12 items of the SRHI include characteristics of habitual responses that may not reflect automaticity per se (i.e., the frequency with which a strategy is used), we also report analyses using the self-report automaticity index (SRBAI) subscale identified by Gardner, Abraham, Lally, and De Bruijn (2012). The SRBAI comprises the four items of the SRHI that reflect the extent to which responses are automatic (e.g., performed outside conscious awareness). Responses on this subscale also proved to be reliable (Cronbach’s alpha = 0.90).

## RESULTS

Participants’ scores were trimmed to three standard deviations of their mean. In addition, a set of Kolmogorov–Smirnov tests were conducted to test whether dependent variables deviated from normality. Self-reported emotional state data (*D* = 0.22, *p* > .05), completion time (*D* = 0.24, *p* > .05), IBIs (*D* = 0.27, *p* > .05) and responses to the self-reported habit index (*D* = 0.22, *p* > .05) did not deviate significantly from normality.

### Effect of ER instructions on self-report emotional state

Self-reported emotional state after participants were exposed to the images depicting body injuries at baseline was compared with that obtained during training session 1 (after 50 trials), training session 2 (after 100 trials) and training session 3 (after 150 trials) using a 2-between (Condition: control instructions vs. ER instructions) × 4-within (Time: baseline, training session 1, training session 2, training session 3) mixed design analysis of variance (ANOVA). There were significant main effects of Time, *F*(3, 45) = 16.24, *p* < .001, and Condition, *F*(1, 15) = 22.30, *p* < .001, that were qualified by a significant interaction between Time and Condition, *F*(3, 45) = 10.92, *p* < .001.

The emotional state of participants allocated to the ER condition differed significantly across the four measurement times, *F*(3, 21) = 33.79, *p* < .001. Specifically they felt less negative during training session 1 (*M* = 6.44, SD = 1.13), *t*(7) = –3.19, *p* < .01, training session 2 (*M* = 6.30, SD = 1.54), *t*(7) = –3.06, *p* < .01, and training session 3 (*M* = 6.45, SD = 1.76), *t*(7) = –3.61, *p* < .01, compared with baseline (*M* = 3.25, SD = 0.90). The emotional state of participants allocated to the control condition did not differ between baseline (*M* = 3.39, SD = 0.95), training session 1 (*M* = 3.96, SD = 0.86), training session 2 (*M* = 3.37, SD = 1.45) and training session 3 (*M* = 3.77, SD = 94), *F*(3, 24) = 0.69, *p* = .56. We also examined the effect of condition (ER vs. control) at each of the measurement times. As expected, there was no significant difference between the ER and control conditions at baseline, *t*(15) = –0.33, *p* = .75. However, participants in the ER condition reported feeling significantly less negative than participants in the control condition at training session 1, *t*(15) = 5.24, *p* < .001, training session 2, *t*(15) = 4.04, *p* < .01, and training session 3, *t*(15) = 4.42, *p* < .01.

### Effect of training on completion time

**Figure 2.**
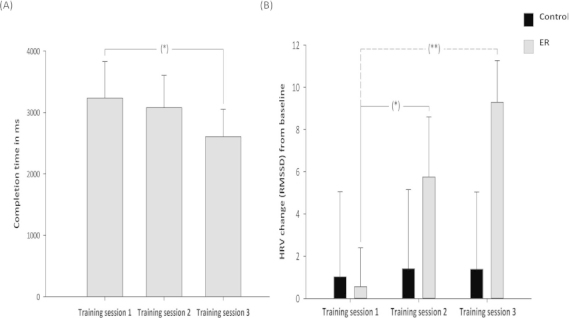
Outcomes during training session 1 (50 trials), training session 2 (100 trials) and training session 3 (150 trials). (A) Time taken in milliseconds (with standard error) by participants in the ER instruction condition to regulate negative affect during each of the three training sessions. (B) Mean change (with standard error) in HRV as a function of instruction condition across training sessions. Change scores were computed subtracting baseline from training. Therefore, positive scores represent increases in HRV reflecting cardiac deceleration. Control = control instruction condition; ER = emotion regulation instruction condition; RMSSD = root mean square of the successive differences. *p < .05. **p < .01.

We examined the time taken for participants to respond to the images depicting body injuries across training sessions. Analyses were conducted separately for each condition (ER vs. control) as the two conditions received different instructions (participants allocated to the ER instruction condition were told to press the “Enter” key only when their emotional response to the images depicting injured body parts subsided, whereas those in the control condition were told to press the “Enter” key every time they were presented with an image depicting a body injury). A repeated measures ANOVA was conducted to examine whether the time that participants in the ER condition took to respond to the images depicting body injuries differed across the three training sessions (50, 100 and 150 trials). There was a significant effect of Training session, *F*(2, 14) = 3.93, *p* < .05 (Figure 2A). This effect was due to a significant decrease in time taken to respond between training session 1 (*M* = 3235, SD = 1683) and training session 3 (*M* = 2607, SD = 1255), *t*(7) = 2.49, *p <* .05, and a trend towards decreased time taken to respond between training session 2 (*M* = 3078, SD = 1493) and training session 3 (*M* = 2607, SD = 1255), *t*(7) = 1.83, *p* = .06. No significant differences in time taken to respond were identified between session 1 (*M* = 3234.5, SD = 1682.89) and session 2 (*M* = 3077.65, SD = 1493.26), *t*(7) = 0.86, *p* = .21. The time taken for participants in the control condition to respond did not differ significantly across training blocks, *F*(2, 16) = 0.20, *p* = .82.

### Effect of training on cardiovascular responses

We conducted a 2-between (Condition: control instructions vs. ER instructions) × 3-within (Training session: first, second and third) mixed design ANOVA to investigate the effect of training on participants’ HRV (Figure 2B). The main effect of Condition was not significant, *F*(1, 15) = 0.84, *p* = .37. However, the main effect of Training session was significant, *F*(2, 30) = 5.33, *p* < .05, as was the interaction between Training session and Condition, *F*(2, 30) = 4.48, *p* < .05.

Simple main effects revealed that this interaction was due to a significant difference in the HRV of participants in the ER condition across training sessions, *F*(2, 14) = 8.42, *p* < .01. Specifically, there was a significant increase in HRV between training session 1 (*M* = 0.56, SD = 5.20) and training session 2 (*M* = 5.76, SD = 8.05), *t*(7) = –3.01, *p* < .05, and between training session 1 and training session 3 (*M* = 9.29, SD =5.59), *t*(7) = –3.69 *p* < .01. Also, there was a trend towards an increase in HRV between training session 2 and training session 3, *t*(7) = –1.55, *p* = .08. No significant difference was identified for the HRV of the control condition across training blocks, *F*(2, 16) = 0.03, *p* = .97. We also examined the effect of condition (ER vs. control) at each of the measurement times. There was no significant difference between the ER and control conditions at training session 1, *t*(15) = –0.10, *p* = .46, and training session 2, *t*(15) = 0.91, *p* = .19. However, participants in the ER condition had significantly higher HRV compared to participants in the control condition at training session 3, *t*(15) = 1.83, *p* < .05.

### Effect of training on emotional experience at follow-up

**Figure 3.**
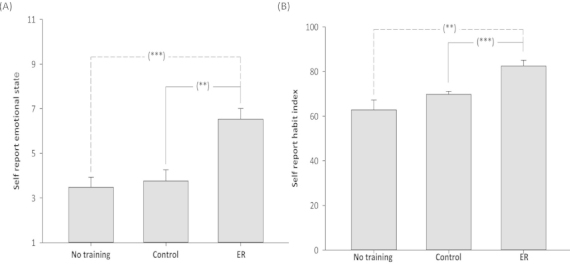
Outcomes at the follow-up session two weeks after the final training session. (A) Self-reported emotional state (with standard error) by Condition at follow-up. Participants indicated to what extent they felt either positive or negative on an 11-point scale anchored by “extremely negative” (1) and “extremely positive” (11). (B) SRHI (with standard error) by Condition at follow-up. Participants were provided with the stem—“Changing the way that I think about negative emotional situations in order to feel better is something”—and then responded to a number of statements (e.g., I do without thinking) on 11-point scales anchored by “strongly disagree” (1) to “strongly agree” (11). Scores could reach a maximum of 132, with high scores reflecting more habitual responses. No training = participation at follow-up only; Control = control instruction condition; ER = emotion regulation instruction condition. ***p < .001.

A 3-between (Condition: ER instruction, control instruction, no training) ANOVA was conducted with participants’ emotional state after watching the follow-up videos as the dependent variable (Figure 3A). The main effect of Condition was significant, *F*(2, 26) = 11.81, *p* < .001. Participants in the ER condition reported feeling significantly less negative (*M* = 6.53, SD = 1.35) than participants in the control condition (*M* = 3.75, SD = 1.51), *t*(15) = 3.96, *p* < .01, and participants in the no-training condition (*M* = 3.48, SD = 1.41), *t*(16) = 4.64, *p* < .001. There was no significant difference in the emotional state of participants in the control and no-training conditions, *t*(17) = 0.41, *p* = .34.

### Effect of training on habitual use of reappraisal at follow-up

A 3-between (Condition: ER instruction, control instruction, no-training) ANOVA was conducted to investigate the extent to which ER had become habitual in the three conditions at follow-up (Figure 3B). There was a significant main effect of Condition on participants responses on the SRHI, *F*(2, 26) = 9.29, *p* < .01. Participants in the ER condition reported being more habitual (*M* = 82.47, SD = 7.06) in their application of ER than participants in the control condition (*M* = 69.83, SD = 4.24), *t*(15) = 4.54, *p* < .001, and participants in the no-training condition (*M* = 62.78, SD = 13.96), *t*(16) = 3.62, *p* < .01. There was no significant difference in the habitual use of ER between participants in the control and no-training conditions, *t*(17) = 1.45, *p* = .17.

We also examined participants’ responses on the automaticity subscale of the SRHI (the SRBAI; Gardner et al., 2012). There was a significant main effect of Condition on participants responses on the SRBAI, *F*(2, 26) = 23.83, *p* < .001. Participants in the ER condition reported being more automatic (*M* = 34.81, SD = 2.07) in their application of ER than participants in the control condition (*M* = 27.21, SD = 2.58), *t*(15) = 7.59, *p* < .001, and participants in the no-training condition (*M* = 24.50, SD = 4.28), *t*(16) = 10.31, *p* < .001. There was no significant difference in the use of ER between participants in the control and no-training conditions, *t*(17) = 2.71, *p* = .24.

## DISCUSSION

The present study investigated whether structured practice could enhance the immediacy and efficiency with which people regulate negative emotions. Participants who completed training in reappraisal showed successful downregulation of negative affect while decreasing the time needed to apply ER and the amount of cardiovascular effort invested in ER. Moreover, participants who completed training were able to regulate their (negative) emotions in the absence of explicit instructions two weeks later, indicating that negative events (in this case, being asked to watch a distressing film) now elicited ER in a spontaneous manner. Participants who completed training also reported being more habitual and automatic in their application of reappraisal. The findings of the present research are consistent with research into the effects of cognitive bias modification on ER, which suggests that structured practice can promote the effective downregulation of negative emotions after the end of an intervention (MacLeod et al., 2009). The findings are also consistent with those of Thiruchselvam, Hajcak, and Gross (2012) who found that cognitive strategies, like the allocation of attention to the non-arousing aspects of the presented negative stimuli, can reduce emotional responses, and more broadly, with research on the beneficial effects of reappraisal (for a review, see Webb et al., 2012). However, the present study expands such findings beyond tasks involving attention allocation to investigate the effects of structured practice in the application of a reappraisal ER strategy. The findings of the present research support those of Schartau et al. (2009), and extend them to demonstrate that practice can promote more automatic ER; an effect that was maintained two weeks later.

The use of physiological measures such as HRV that are sensitive to effort invested during ER, along with measures of the extent to which individuals regulated their emotions in a more automatic fashion (namely the SRBAI), offered insights into the relationship between structured practice in ER and the efficiency of ER. Overall, the findings showed that structured practice led to increases in HRV during ER, reflecting decreased cardiovascular effort. In turn, participants receiving structured practice reported being more habitual and automatic in their use of reappraisal during the follow-up compared with participants in the control and no-training conditions. Taken together, these findings suggest that physiological measures can be used to measure changes in the efficiency with which cognitive processes such as ER are implemented. Although physiological measures may not be practical in all contexts, they have the advantage of being non-reactive and sufficiently sensitive to changes elicited over relatively short time periods (for a review of the use of physiological measures in research on emotions, see Mauss & Robinson, 2009).

### Practical applications

The short duration of the present intervention and the minimal need for one-to-one contact suggest that similar protocols could be implemented online or developed as part of computerised self-help packages. This kind of application could offer people a structured approach to promoting effective and efficient ER and therefore help them to gain control of problematic emotions while avoiding the drain on resources that is associated with effortful forms of ER. For example, professionals who are subjected to continued stress, such as emergency room staff (Jensen et al., 2013), are likely to benefit from the increased resilience that is afforded by more automatic forms of ER. The use of effortful forms of ER under these circumstances would not permit sustained regulation of emotional responses. More automatic forms of ER, on the other hand, use fewer self-regulatory resources, thereby allowing prolonged regulation of emotional responses. Such applications of the current training paradigm are in accord with researches examining the promotion of emotional resilience. For example, Stallard et al. (2005) reported that the provision of a cognitive behavior therapy programme incorporating training in ER led to decreased anxiety measures thereby promoting emotional resilience.

### Limitations and future research

Due to the intensity of the present research paradigm, requiring repeated training and assessment over a number of days, our sample size was relatively small (*n* = 27 participants, across three conditions), though comparable to those used in similar experimental designs (Schneider & Shiffrin, 1977a, 1977b; Mahdavi-Haji, Mohammadkhani, & Hahtami, 2011; Webb, Sheeran, & Luszczynska, 2009). The relative small sample of the present research meant that we were unable to examine individual differences in responses to training. Future research should, however, seek to replicate the present findings among larger, more diverse samples that would allow the examination of individual differences. Given that training effects can display context sensitivity (Reder & Klatzky, 1994), subsequent research should also measure responses to real-world events in order to examine whether the effects of structured practice in ER are transferable to settings outside the laboratory. Finally, future research should examine whether structured training can aid ER using a more varied set of stimuli and instructions. Our finding that ER at follow-up was relatively habitual suggests that effects should be maintained over longer follow-up periods. However, it may also be that additional strategies or reminders are required to maintain effects over time.

## CONCLUSION

In summary, the present research suggests that structured practice in the application of reappraisal can foster efficient and fast control of emotional responses. Such effects are sustained two weeks following training and become relatively more automatic. These findings are likely to hold practical implications for promoting emotional resilience for professionals who are subject to ongoing stress.
